# Reply to Suetsugu and Johnson: Bird dispersal of berry-like yam bulbils is consistent with Batesian mimicry under low receiver cost

**DOI:** 10.1073/pnas.2604887123

**Published:** 2026-03-19

**Authors:** Guillaume Chomicki, Zhi Chen, Ying Li, Xingrong Peng, Gao Chen

**Affiliations:** ^a^Department of Biosciences, Durham University, Durham DH1 3LE, United Kingdom; ^b^State Key Laboratory of Phytochemistry and Natural Medicines, Kunming Institute of Botany, Chinese Academy of Sciences, Kunming 650204, China; ^c^Yunnan Key Laboratory for Integrative Conservation of Plant Species with Extremely Small Populations, Kunming Institute of Botany, Chinese Academy of Sciences, Kunming 650204, China; ^d^University of Chinese Academy of Sciences, Beijing 100049, China; ^e^Yunnan Key Laboratory of Plant Diversity and Biogeography, Kunming Institute of Botany, Chinese Academy of Sciences, Kunming 650201, China

Suetsugu and Johnson (1) question whether bird dispersal of *Dioscorea melanophyma* bulbils (2) constitutes Batesian mimicry or generalized food deception.

Under generalized food deception, signals exploit innate sensory biases and are untraceable to particular model species (3). Although *D. melanophyma* bulbils mimic a guild of black-fruited species rather than a single model, the spectral precision of this resemblance argues against generalized food deception. Chromatic contrast between bulbils and 15 sympatric berry species fell below one just-noticeable difference (JND < 1) in a UV-sensitive avian vision model (2). Crude resemblance would suffice under innate bias; this precision is expected under multifarious Batesian mimicry (4). Innate fruit color preferences are overridden by experience in wild adults (5); the relevant receivers are experienced frugivores shaped by the local berry community.

Suetsugu and Johnson question model dependence, noting that peak bulbil removal when berries are scarce appears contradictory. Bulbil display (July through May) overlaps extensively with berry fruiting, providing ample opportunity for birds to learn berry traits as rewarding. In cafeteria trials, wild-caught adults approached bulbils at high rates, yet postcontact discrimination was very high: Berries were ingested on every visit, whereas bulbils were rejected in over 92% of approaches (2). Because the spectral match (JND < 1) renders bulbils and berries perceptually identical before contact, approach to bulbils is an incidental by-product of a search image maintained by rewarding berry encounters. This is model dependence: Bulbil deception functions because genuine berries sustain the learned response it exploits. That bulbil approaches were more frequent when fewer berries were available reflects intensified foraging under scarcity, not weakened model dependence.

What differs from protective Batesian mimicry is the strength of frequency dependence. In protective systems, a receiver error—consuming a toxic model—can be lethal (6), generating strong selection to discriminate and pronounced negative frequency dependence on mimics. Here, the cost of approaching a nonrewarding bulbil is trivial: seconds of handling, no toxicity. Accordingly, Anderson and Johnson (7) found no evidence of learned avoidance in a floral mimicry system where receiver costs were limited to wasted handling time. Very high (92%) postcontact rejection yet continued precontact approach is consistent: The spectral match renders precontact discrimination perceptually impossible, and the negligible cost of erroneous approach reduces selection pressure to evolve reliance on supplementary precontact cues.

This exchange highlights a terminological tension. Batesian mimicry was defined for defensive systems where the receiver is repelled (6) and later extended to pollination, where the receiver is attracted (8), a usage itself contested (9). The core architecture is shared: A nonrewarding species co-opts signals from rewarding models to deceive receivers. What changes across contexts is receiver cost, which modulates the strength of frequency dependence (7, 10). A framework in which receiver cost is an explicit variable would help predict mimicry dynamics across defensive, pollination, and dispersal systems. We welcome the co-occurrence tests proposed by Suetsugu and Johnson.
